# MicroRNA-122 Inhibits the Production of Inflammatory Cytokines by Targeting the PKR Activator PACT in Human Hepatic Stellate Cells

**DOI:** 10.1371/journal.pone.0144295

**Published:** 2015-12-04

**Authors:** Masato Nakamura, Tatsuo Kanda, Reina Sasaki, Yuki Haga, Xia Jiang, Shuang Wu, Shingo Nakamoto, Osamu Yokosuka

**Affiliations:** 1 Department of Gastroenterology and Nephrology, Chiba University, Graduate School of Medicine, Chiba, 260–8677, Japan; 2 Department of Molecular Virology, Chiba University, Graduate School of Medicine, Chiba, 260–8677, Japan; Saint Louis University, UNITED STATES

## Abstract

MicroRNA-122 (miR-122) is one of the most abundant miRs in the liver. Previous studies have demonstrated that miR-122 plays a role in inflammation in the liver and functions in hepatic stellate cells (HSCs), which reside in the space of Disse. Here, we showed that the transient inhibition of PKR-activating protein (PACT) expression, by miR-122 or siRNA targeting of PACT, suppressed the production of proinflammatory cytokines, such as interleukin (IL)-6, monocyte chemoattractant protein-1 (MCP-1) and IL-1β, in human HSC LX-2. Sequence and functional analyses confirmed that miR-122 directly targeted the 3′-untranslated region of PACT. Immunofluorescence analysis revealed that miR-122 blocked NF-κB-nuclear translocation in LX-2 cells. We also showed that conditioned medium from miR-122-transfected LX-2 cells suppressed human monocyte-derived THP-1 cell migration. Taken together, our study indicates that miR-122 may downregulate cytokine production in HSCs and macrophage chemotaxis and that the targeting of miR-122 may have therapeutic potential for preventing the progression of liver diseases.

## Introduction

Hepatic stellate cells (HSCs) are located in the space of Disse, between the basolateral surfaces of hepatocytes and the anti-luminal sides of sinusoidal endothelial cells. HSCs are able to interact with neighboring cells, such as hepatocytes and bone marrow-derived cells, through the intercellular transport of soluble mediators, cytokines and chemokines [1], although they are also known to be one of the major contributors to the progression of hepatic fibrosis.

During liver injury, HSCs are activated and differentiated into alpha smooth muscle actin-expressing contractile myofibroblasts [2]. Activation of HSCs increases fibrogenesis with the regulation of inflammation and immune response, and the alteration of matrix degradation [2]. Hepatic fibrosis is the consequence of an imbalance between the production and degradation of the extracellular matrix [3].

Toll-like receptors (TLRs) are pattern-recognition receptors that contribute to innate and adaptive immunity in humans. Several studies have shown that TLR4 signaling is involved in the pathogenesis of various liver diseases, such as alcoholic liver disease (ALD), non-alcoholic steatohepatitis (NASH) and chronic hepatitis C [4–6]. Especially, gut-derived LPS-activated TLR4 signaling contributes to inflammation and fibrosis of the liver [7]. Intact TLR4 signaling has been reported in HSCs [7]. In activated HSCs, the production of various cytokines and chemokines has also been observed [1,8]. The binding of lipopolysaccharide (LPS), a structural component unique to gram-negative bacteria, to TLR4 stimulates the MyD88-dependent and MyD88-independent signaling pathways, which are involved in the production of proinflammatory cytokines and interferon, respectively [9]. At least 3 major transcriptional complexes, including nuclear factor (NF)-κB, activator protein (AP)-1 and interferon regulatory factors (IRFs), are involved in TLR4 signaling in HSCs [7]. Activation of these transcription factors leads to the production of proinflammatory cytokines (TNF-α, IL-1β and IL-6), chemotactic cytokines [monocyte chemoattractant protein-1 (MCP-1)/chemokine (C-C motif) ligand 2 (CCL2) and macrophage migration inhibitory factor (MIF)], proinflammatory proteins [inducible nitric oxide synthase (iNOS)], and reactive oxygen species (ROS) [7].

It is well known that double-stranded RNA (dsRNA)-activated serine-threonine protein kinase (PKR), a latent protein kinase, mediates the antiviral activities of interferon. PKR is activated by dsRNA and inhibits protein synthesis by phosphorylating eukaryotic translation initiation factor-2α (eIF2α) in virally infected cells [10]. In addition to its translational regulatory function, PKR directly phosphorylates IκB and regulates the NF-κB pathway [11]. PKR activating protein (PACT) [protein kinase, interferon-inducible dsRNA-dependent activator (PRKRA)] can bind to the PKR kinase domain and acts as a cellular activator of PKR in the absence of dsRNA [12]. PACT is an important molecule for the production of interferon and cytokines [12–14].

Endogenous microRNAs (miRs) are non-coding RNAs of 19–23 nucleotides in length. MiRs are post-transcriptional regulators that bind to the 3′-untranslated region (3′-UTR) of target gene mRNAs, resulting in silencing of their functions by cleavage mRNAs or inhibition of the translation [15]. MiR-122 represents approximately 70% of the total miRs in the liver [16,17]. It has been reported that miR-122 is associated with lipid metabolism, stress response and hepatitis C virus (HCV) replication [18]. MiR-122 also plays a role in hepatic inflammation [19]. In rats, miR-122 is constitutively expressed in HSCs, and its expression level is decreased in activating HSCs, suggesting its importance in hepatic fibrosis [20]. Yet, the role of miR-122 in HSCs on hepatic inflammation is not well known. The present study showed that miR-122 inhibits the production of proinflammatory cytokines by targeting PACT in human HSCs. Our study also revealed that miR-122 in HSCs might be an important regulator of hepatic inflammation and could have therapeutic potential for preventing the progression of liver diseases.

## Materials and Methods

### Cells and Transfection

A spontaneously immortalized human hepatic stellate cell line, LX-2 (kindly provided by Prof. Friedman, S. L., Mount Sinai Medical School, NY)[21], was maintained in Dulbecco’s Modified Eagle’s Medium (DMEM) (Sigma, St. Louis, MO) supplemented with 100 U/mL penicillin, 100 μg/mL streptomycin (Gibco BRL, Gaithersburg, MD) and 10% or 1% fetal calf serum (FCS) (Gibco BRL) in a humidified 5% CO_2_ incubator at 37°C. A human acute monocytic leukemia cell line, THP-1 (purchased from the Japanese Collection of Research Bioresources Cell Bank, Ibaraki, Osaka, Japan; No. JCRB0112)[22], was maintained in Roswell Park Memorial Institute (RPMI) 1640 medium (Sigma) with 10% FCS without antibiotics. A primary human hepatic stellate cell line, HHSteC, was purchased from ScienCell Research Laboratories (Carlsbad, CA) and maintained in Stellate Cell Medium (ScienCell Research Laboratories) with 2% FCS plus stellate cell growth supplement, 100 U/mL penicillin and 100 μg/mL streptomycin (ScienCell Research Laboratories).

Hsa-miR-122-5p mimic (miR-122) and control miR (miR-C) were purchased from Life Technologies (Tokyo, Japan). siRNA against PACT (si-PACT) and control siRNA (si-C) were obtained from Santa Cruz Biotechnology (Santa Cruz, CA). Transfections were performed with 50 nM miR-122, 50 nM miR-C, 20 nM si-PACT, or 20 nM si-C using Effectene Transfection Reagents (Qiagen, Hilden, Germany) according to the manufacturer’s protocol.

### LPS Stimulation

At twenty-four hours after incubation in DMEM with 1% FCS, LX-2 cells were stimulated with 100 ng/mL LPS (Imgenex, San Diego, CA) for 24 hours.

### RNA Quantitation

Total RNA was isolated with QIA Shredder and RNeasy Mini kits (Qiagen). Complementary DNA (cDNA) was synthesized with random hexamers and oligo dT primers using a PrimeScript RT Reagent kit (Takara BIO, Otsu, Shiga, Japan). Quantitative RT-PCR was performed using the cDNA for RNA quantitation with Power SYBR Green PCR Master Mix and a StepOne Real-Time PCR system (Applied Biosystems, Foster City, CA). Glyceraldehyde 3-phosphate dehydrogenase (GAPDH) was used as an endogenous control. Data analysis was performed by the 2^-ddCt^ (comparative cycle threshold) methods. Primers for IL-1β, IL-6, GAPDH, MCP-1, PACT and TNF-α were synthesized by Sigma Aldrich Japan. The sequences of these primers have been previously described [23–25].

### MiR Quantitation

Total RNA was isolated with QIA Shredder and miRNeasy Mini kits (Qiagen). cDNA was synthesized using a TaqMan MicroRNA Reverse Transcription kit (Applied Biosystems) with miRNA-specific primers. For miR quantitation, real-time PCR was conducted using gene-specific TaqMan assay kits for miR-122 and U6 snRNA with a StepOne Real-Time PCR System (Applied Biosystems). U6 snRNA was used as an endogenous control. Data analysis was performed by 2^-ddCt^ methods. Prediction of miR targets was performed with TargetScanHuman (http://www.targetscan.org/).

### PCR Array

Gene expression profiling was performed using RT^2^ profiler PCR arrays for the human TLR signaling pathway (Qiagen) following the manufacturer's instructions. Briefly, 1 μg of RNA was reverse-transcribed with a RT^2^ First Strand kit (Qiagen) using a Takara PCR thermal cycler (TP3000, Takara), and PCR was performed with RT^2^ SYBR Green ROX qPCR Master Mix (Qiagen) using an ABI PRISM 7300 (Applied Biosystems). Genes were annotated using Entrez Gene (NCBI, Bethesda, MD). Eighty-four genes and functional gene groupings are available on this website (http://www.sabiosciences.com/rt_pcr_product/HTML/PAHS-018Z.html).

Gene expression was normalized to five internal controls (β-2-microglobulin, hypoxanthine phosphoribosyltransferase 1, ribosomal protein large P0, GAPDH and β-actin) to determine the fold-changes in gene expression between the test sample (miR-122-transfected-LX-2) and the control sample (miR-C-transfected-LX-2) by the 2^-ddCt^ methods. These PCR arrays were performed as previously described [6]. Data were analyzed with RT^2^ Profiler PCR Array Data Analysis software (http://pcrdataanalysis.sabiosciences.com/pcr/arrayanalysis.php).

### Western Blot Analysis

Cell lysates were collected in sodium dodecyl sulfate (SDS) sample buffer. After sonication, samples were subjected to electrophoresis on 5–20% polyacrylamide gels and transferred to polyvinylidene difluoride membranes (ATTO, Tokyo, Japan). The membranes were probed with specific antibodies to PACT, phosphorylation of PKR (Thr451), PKR (Santa Cruz Biotechnology), phosphorylation of IκBα (Ser32), IκBα (Cell Signaling Technology, Boston, MA) or GAPDH (Santa Cruz Biotechnology) for internal control. After a washing step, the membranes were incubated with secondary HRP-conjugated antibodies. Signals were detected with an ECL (GE Healthcare Japan, Tokyo, Japan) and scanned using an LAS-4000 image analyzer and Image Gauge (version 3.1) (Fuji Film, Tokyo, Japan) [6].

### ELISA

Conditioned media were collected and stored at -30°C until use. Human IL-1β, IL-6 and MCP-1 were measured using a Pink-ONE Cytokine-ELISA kit (Koma Biotech, Seoul, Korea) according to the manufacturer’s protocols. Briefly, conditioned media were added to plates and incubated at room temperature for 4 hours, followed by incubation with biotinylated monoclonal antibodies. Avidin-conjugated peroxidase was added to the plates, and enzyme activity was detected with an ELISA plate reader (iMark microplate reader, Bio-Rad, Hercules, CA)[24]. The sensitivities of human IL-1β, IL-6 and MCP-1 for these ELISA kits were 4–250 pg/mL, 32–2,000 pg/mL and 16–1,000 pg/mL, respectively.

### Luciferase Assay

A total of 3 x 10^5^ LX-2 cells were co-transfected with either 0.1 μg PACT 3´-UTR (wild) or PACT 3´-UTR (mutant) reporter clones for miRNA target validation (Origene, Rockville, MD) and 10 ng phRluc-TK vector (Promega, Madison, WI), which is a transfection efficiency internal control plasmid expressing *Renilla reniformis* luciferase, and 50 nM miR-122. The 3´-UTR of PACT was cloned downstream of the firefly luciferase gene. An internal ribosomal entry site (IRES) is located upstream of the firefly luciferase gene. When the miR-122/RNA-induced silencing complex (RISC) binds to 3´-UTR of PACT mRNA, firefly luciferase activity is reduced. If there is no interaction between miR-122 and 3´-UTR of the PACT mRNA, luciferase activity remains high. Cells were harvested at 48 hours after transfection, and luciferase activity was measured using a Picagene Dual Sea Pansy System (Toyo Ink, Tokyo, Japan) [6]. Firefly and sea pansy luciferase activities were measured as relative light units with a luminometer (Luminescencer-JNR II AB-2300, ATTO).

### Immunofluorescence Study

A total of 2 x 10^5^ LX-2 cells were seeded on 35-mm dishes at 24 hours before transfection. Then, the cells were transfected with miR-122, miR-C, si-PACT or si-C. Thirty-six hours post-transfection, the cells were treated with 100 ng/mL LPS or 1 x 10^5^ U/mL IFN-α. Two hours later, they were fixed with 3.7% formaldehyde for 30 min, followed by blocking with 3% bovine serum albumin for 1 hour. They were then incubated with an NF-κB p65 antibody (Cell Signaling) for 16 hours at 4°C. Next, they were washed and incubated with anti-rabbit immunoglobulin secondary antibody conjugated to Alexa Fluor 555 (Cell Signaling) for 1 hour at room temperature. Nuclear staining was performed with Hoechst 33342, trihydrochloride, and trihydrate (Molecular Probes, Eugene, OR). Finally, the cells were washed and mounted for confocal microscopy (ECLIPSE TE 2000-U, Nikon, Tokyo, Japan), and the images were superimposed digitally to allow fine comparisons [25].

### Chemotaxis Assay

A chemotaxis assay was performed with polycarbonate membrane inserts with 5-μm pore size using a CytoSelect 24-well cell migration assay kit (Cell Biolabs, San Diego, CA) according to the manufacturer’s instructions. In brief, 5 x 10^5^ THP-1 cells were placed in the upper chamber, and conditioned medium from LPS-stimulated LX-2 cells was added to the lower chamber. After 3 hours of incubation in a cell culture incubator, migratory cells that had detached from the bottom side of the inserted membrane and cells that had migrated into the lower chamber were combined. The migratory cells were lysed and quantified using CyQuant GR Fluorescent Dye (Invitrogen, Carlsbad, CA). Fluorescence was measured using a fluorescence plate reader (TriStar^2^ multimode reader LB942, Berthold Technologies, Tokyo, Japan) with a 480/535 nm filter set. These assays were performed in triplicate.

### PKR Inhibitor Treatment

The PKR inhibitor C16 (Calbiochem, Merck Millipore, Billerica, MA) was dissolved in dimethyl sulfoxide (DMSO) following the manufacturer's instructions [26]. The PKR inhibitor or vehicle was added to LX-2 cells at 1 hour prior to LPS stimulation at the indicated concentrations. Then, the cells were treated with LPS for 24 hours in the presence or absence of the PKR inhibitor.

### MTS Assay

To evaluate cell viability, MTS assays were performed using the CellTiter Aqueous One Solution Proliferation Assay (Promega) according to the manufacturer’s instruction [6].

### Statistical Analysis

Statistical analysis was performed by Student’s t-test. A *p*<0.05 value was considered statistically significant. Data are expressed as mean ± standard deviations (SD).

## Results

### LX-2 cells produce proinflammatory cytokines after LPS stimulation

**Fig 1:**
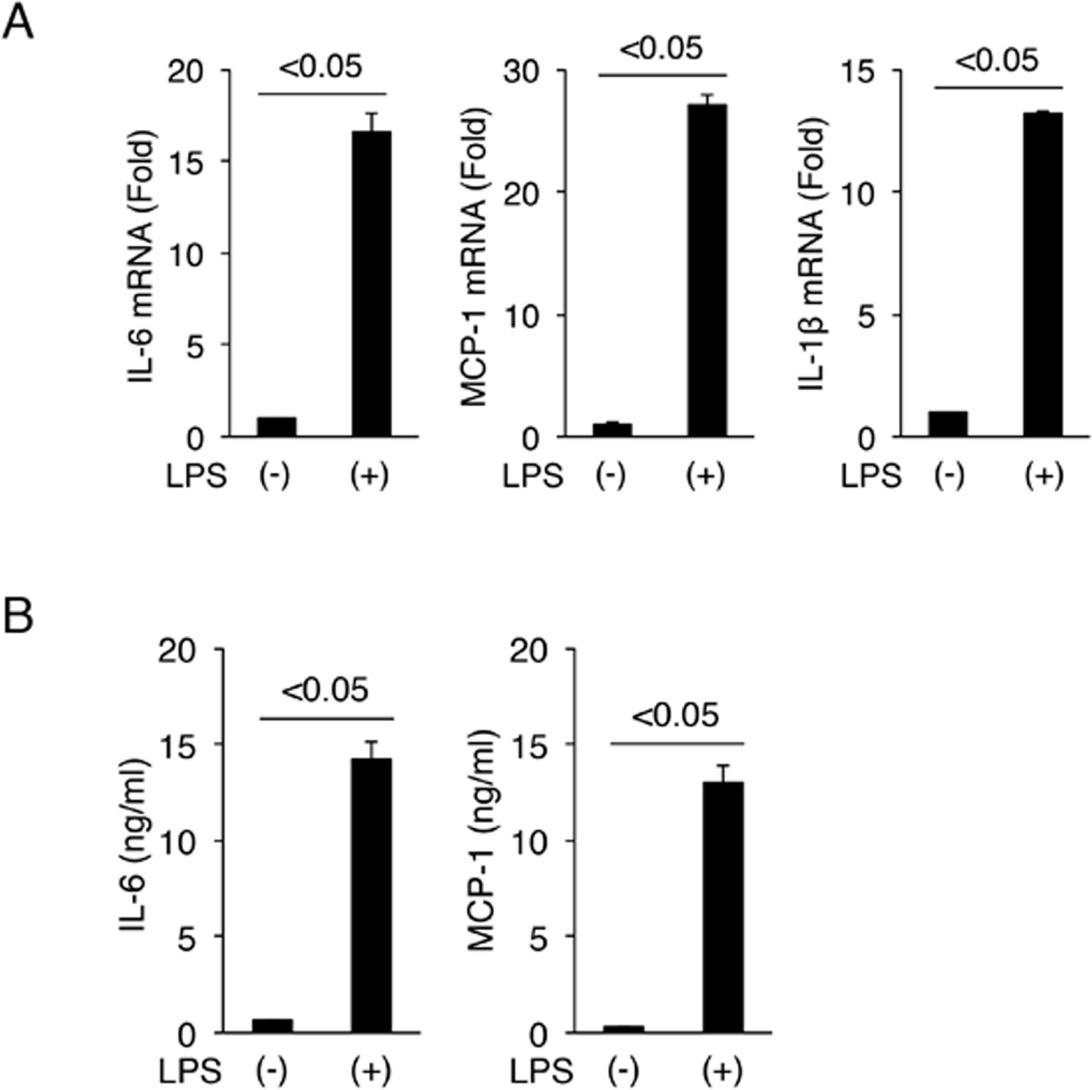
Cytokine production in LX-2 cells. (A) Effects of LPS stimulation on the expression of cytokine mRNA. LX-2 cells were treated with or without 100 ng/mL LPS for 24 hours. Gene expression levels of IL-6, MCP-1 and IL-1β were examined by real-time RT-PCR. GAPDH was used for normalization. (B) Effects of LPS stimulation on the expression of cytokines at protein level. IL-6 and MCP-1 protein levels were determined by ELISA. Results are expressed as mean ± SD. Minimum three replicates were performed for each set of experiments to compile the data as presented.

First, we examined whether human HSC LX-2 responds to TLR4 ligand LPS. The expression levels of IL-6, MCP-1 and IL-1β mRNA were examined and were found to be upregulated in the LX-2 cells treated with LPS for 24 hours (by approximately 16.6-fold, 27.1-fold, and 13.2-fold, respectively) (Fig 1A). We also confirmed the protein levels of IL-6 and MCP-1 (Fig 1B). TNF-α is an important cytokine in the liver as well and its expression is triggered by LPS in HSCs. TNF-α mRNA expression was also upregulated (94.6-fold, compared to basal level) at 2 hours after the addition of LPS, but it was decreased to basal level at 24 hours post-stimulation. Together, these results suggest that LPS induced inflammatory cytokines in LX-2 cells, supporting the concept that LX-2 cells possess a functional TLR4 signaling pathway [27].

### MiR-122 inhibits cytokine production in LX-2 cells

**Fig 2:**
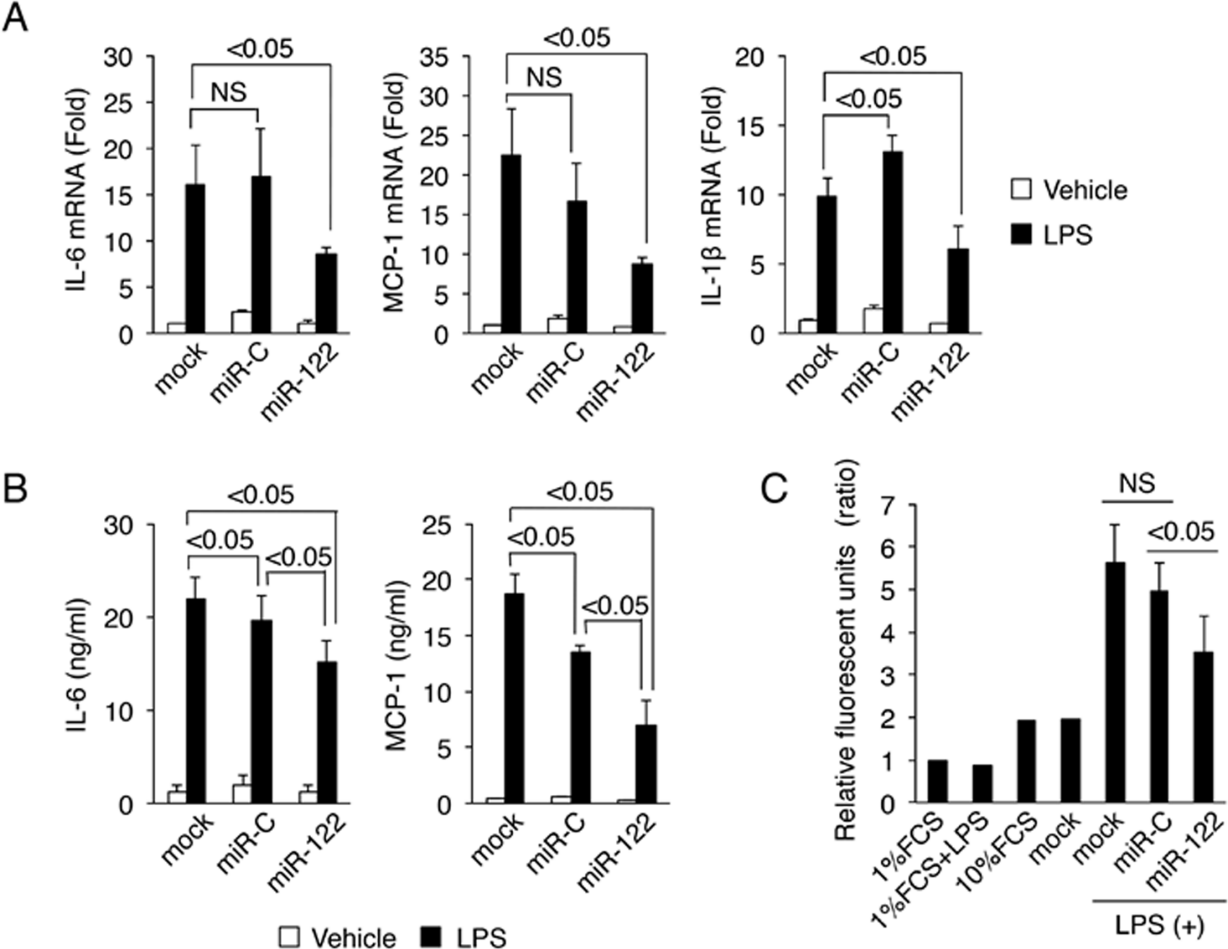
MiR-122 inhibits cytokine production in LX-2 cells and suppresses the migration of THP-1 cells. (A) Effects of miR-122 on the expression of cytokine mRNA. LX-2 cells were transfected with mock, 50 nM control miRNA (miR-C) or 50 nM miR-122. After incubation in DMEM with 1% FCS, the cells were treated with 100 ng/mL LPS for 24 hours. Cellular RNAs were isolated, and the expressions of IL-6, MCP-1 and IL-1β mRNA levels were examined by real-time RT-PCR. GAPDH mRNA was used for normalization. (B) Effects of miR-122 on the expression of cytokines at protein level. Conditioned medium was collected from LPS-stimulated LX-2 cells, and ELISA was performed to assess IL-6 and MCP-1 expression. (C) Migration of the human monocyte cell line THP-1 was suppressed by conditioned media from miR-122-transfected LX-2 cells. A total of 5 x 10^5^ THP-1 cells were placed in the upper chamber, and conditioned medium from LPS-stimulated LX-2 cells transfected with miR-C or miR-122 was added to the lower chamber. After 3 hours of incubation, THP-1 cells that migrated toward the lower chamber were detected by fluorescent dye. Relative fluorescence units are indicated (*vs*. 1%FCS). All results are shown as mean ± SD. Minimum three replicates were performed for each set of experiments to compile the data as presented.

We next examined whether miR-122 could inhibit the production of inflammatory cytokines induced by LPS in HSCs. IL-6, MCP-1 and IL-1β mRNA expression levels in the LX-2 cells transfected with miR-122 were significantly inhibited, compared with those in the LX-2 cells transfected with miR-C (Fig 2A). The modulation of IL-6 and MCP-1 by miR-122 in conditioned medium was also verified by ELISA. Our results demonstrated that IL-6 and MCP-1 were reduced in conditioned medium from the miR-122-transfected LX-2 cells, compared with control (Fig 2B). IL-1β in conditioned medium from the untransfected, miR-C-transfected or miR-122-transfected LX-2 cells was undetectable, 4.55 pg/mL or undetectable, respectively.

### Conditioned media from miR-122-transfected LX-2 cells suppress human monocyte-derived THP-1 cell migration

We examined the functional role of the miR-122-mediated inhibition of inflammatory cytokine expression. Because MCP-1 functions as a chemoattractant cytokine for monocytes, macrophages and Kupffer cells [28], which seem to contribute to inflammation in the liver [29], we focused on cell migration of the human monocyte-derived cell line THP-1. We performed *in vitro* migration assays to determine whether conditioned media from miR-122-transfected LX-2 cells could suppress the migration of THP-1 cells. For this experiment, conditioned media from LX-2 cells transfected with miR-122 or miR-C were evaluated. The migration of THP-1 cells was markedly decreased (~68%) when conditioned media from LX-2 cells transfected with miR-122 was used (Fig 2C). The number of migrated THP-1 cells was 31,700/well, 28,000/well or 19,800/well when conditioned medium from LX-2 cells transfected with mock, miR-C or miR-122, respectively, was used. These data suggest that the inhibition of inflammatory cytokine production together with miR-122 in HSCs inhibits the migration of monocytes and monocyte-derived cells, supporting the concept that miR-122 prevents hepatic inflammation by inhibiting cytokine production and the recruitment of immune cells to the liver.

### MiR-122 modulates TLR signaling pathway in LX-2 cells

**Fig 3:**
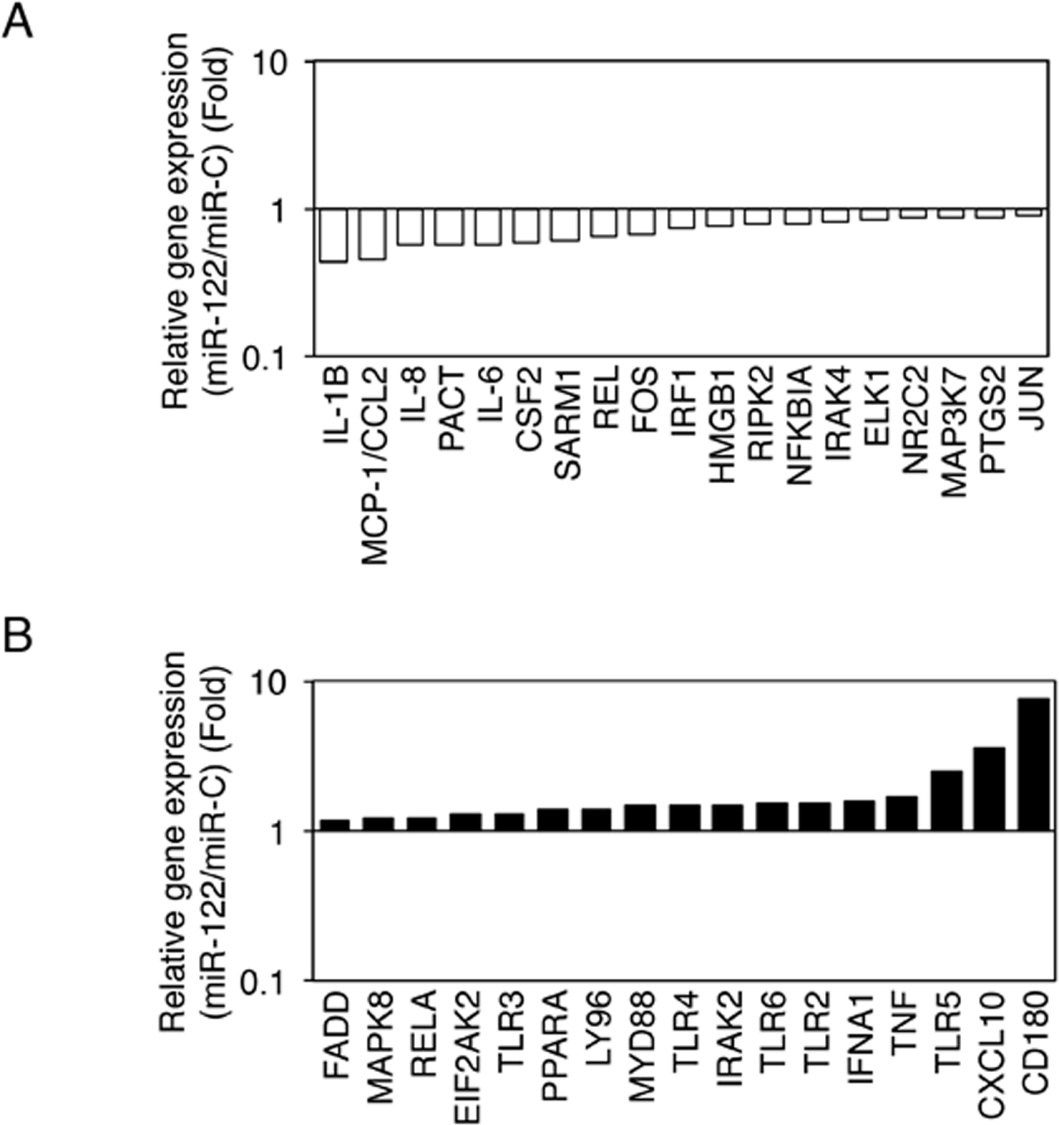
MiR-122 modulates the expression of genes related to TLR and its associated signaling pathway in LX-2 cells. (A) Downregulated genes in miR-122-transfected LX-2 cells compared with miR-C-transfected LX-2 cells. (B) Upregulated genes in miR-122-transfected LX-2 cells compared with miR-C-transfected LX-2 cells. RNAs were isolated from LX-2 cells transfected with miR-122 or miR-C, and PCR array analysis was performed to assess the expression of TLR and its related genes. Real-time PCR array analysis was performed, and the results were analyzed using RT^2^ Profiler PCR Array Data Analysis software. IL, interleukin; MCP-1, monocyte chemoattractant protein-1; CCL2, chemokine (C-C motif) ligand 2; PACT, PKR-activating protein; CSF2, colony-stimulating factor 2; SARM1, sterile alpha and TIR motif containing 1; REL, V-rel reticuloendotheliosis viral oncogene homolog; Fos, FBJ murine osteosarcoma viral oncogene homolog; IRF1, interferon regulatory factor 1; HMGB1, high mobility group box 1; RIPK2, receptor-interacting serine-threonine kinase 2; NFKB1A, nuclear factor of kappa light polypeptide gene enhancer in B cells inhibitor alpha; IRAK, interleukin-1 receptor-associated kinase; ELK1, member of the ETS oncogene family; NR2C2, nuclear receptor subfamily 2, group C, member 2; MAP3K, mitogen-activated protein kinase kinase kinase; PTGS2, prostaglandin-endoperoxide synthase 2; JUN, Jun proto-oncogene; FADD, Fas-associated via death domain; MAPK, mitogen-activated protein kinase; EIF2AK2, eukaryotic translation initiation factor 2-alpha kinase 2; TLR, Toll-like receptor; PPARA, peroxisome proliferator-activated receptor alpha; LY96, lymphocyte antigen 96; MYD88, myeloid differentiation primary response gene (88); IFNA1, interferon, alpha 1; TNF, tumor necrosis factor; CXCL10, chemokine (C-X-C motif) ligand 10; CD180, CD180 molecule. Minimum three replicates were performed for each set of experiments to compile the data as presented.

To further advance mechanistic insights into the role of miR-122 in innate immunity, including its effects on cytokines and the TLR signaling pathway, we assessed the gene expression profile of LX-2 cells. We performed a pathway-specific PCR array to identify miR-122 target genes in miR-122-transfected LX-2 cells compared with miR-C-transfected LX-2 cells (Fig 3A and 3B). Out of 84 TLR-signaling pathway-associated genes, 19 genes (22.6%) were downregulated by 1.17-fold or greater in miR-122-transfected LX-2 cells compared with miR-C-transfected LX-2 cells in the presence of LPS-stimulation (Fig 3A; n = 3, P < 0.05), and 17 genes (20.3%) were upregulated by 1.17-fold or greater in miR-122-transfected LX-2 cells compared with miR-C-transfected LX-2 cells in the presence of LPS-stimulation (Fig 3B; n = 3, P < 0.05). The inhibition of several proinflammatory cytokines, such as IL-1β, MCP-1, IL-8 and IL-6, was observed in the LX-2 cells transfected with miR-122 (Fig 3A).

### MiR-122 directly downregulates PACT expression

In general, miRNAs inhibit gene expression; therefore, we focused on genes downregulated by miR-122. In particular, PCR array analysis showed that miR-122 downregulated PACT mRNA expression (0.56-fold; Fig 3A), and TargetScanHuman revealed that PACT mRNA is a putative target of miR-122.

**Fig 4:**
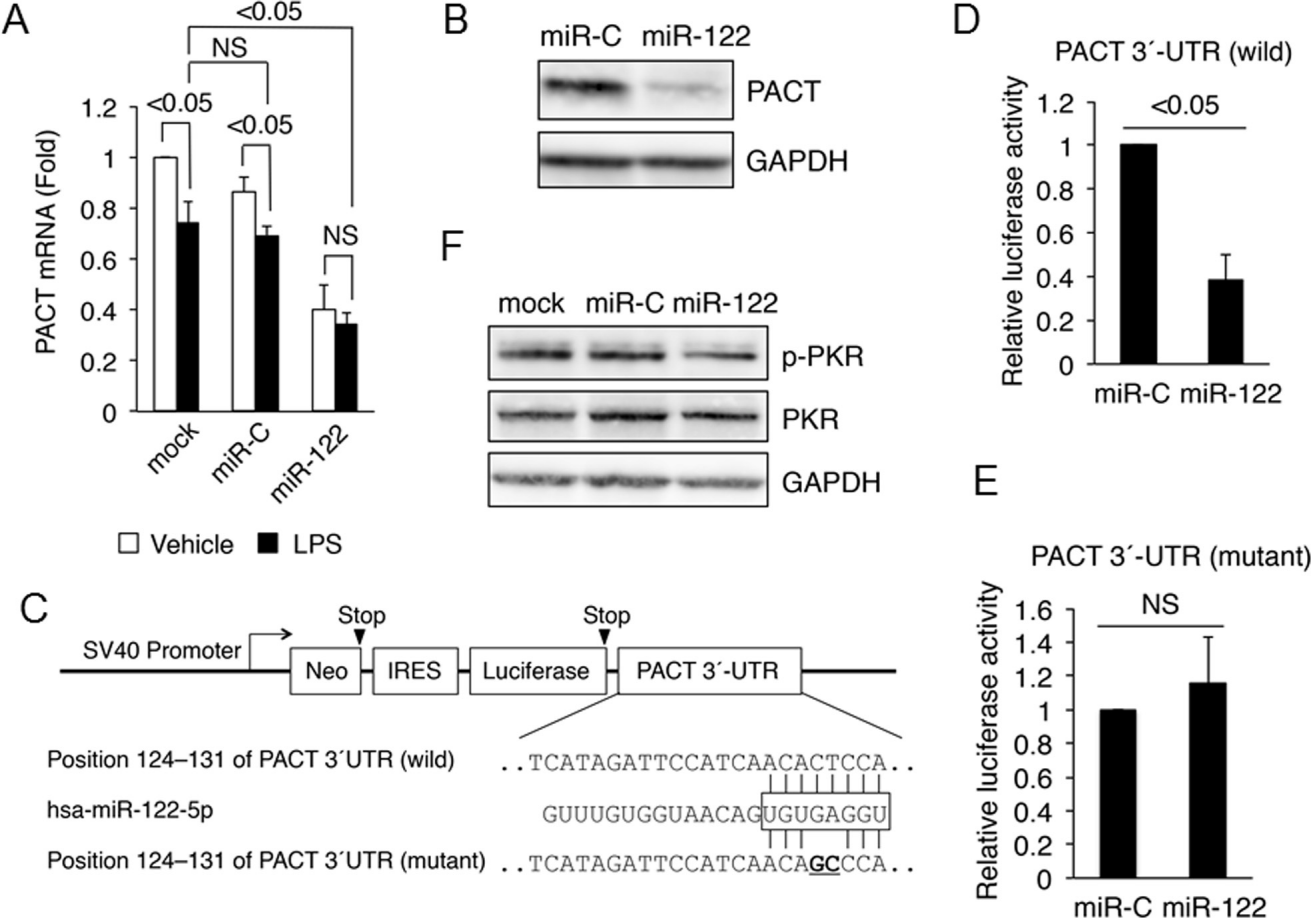
MiR-122 directly inhibits PACT expression in LX-2 cells. (A) MiR-122 inhibits PACT mRNA expression. LX-2 cells were transfected with miR-122 or control miR-C. After incubation in DMEM with 1% FCS for 24 hours, the cells were treated with 100 ng/mL LPS for 24 hours. PACT mRNA was examined by real-time RT-PCR. GAPDH mRNA was used for normalization. (B) MiR-122 inhibits PACT protein expression. Lysates from transfected LX-2 cells were immunoblotted with antibodies against PACT or GAPDH. GAPDH was used as internal control. (C) Nucleotide homology among luciferase constructs of PACT 3´-UTR (wild), PACT 3´-UTR (mutant) and miR-122 (hsa-miR-122-5p). The white rectangle in hsa-miR-122-5p indicates the seed sequence. The nucleotide substitutions in the seed sequence of PACT 3´-UTR are shown in boldface. (D) Inhibition of PACT 3´-UTR (wild) luciferase activity by miR-122. (E) No inhibition of PACT 3´-UTR (mutant) luciferase activity by miR-122. LX-2 cells were transfected with a wild-type or mutant PACT 3´-UTR plasmid and phRluc-TK with miR-122 or miR-C. After 48 hours, luciferase activity was measured using a dual luciferase assay system. Relative luciferase activities (*vs*. miR-C) are shown. The results are expressed as mean ± SD. NS, no significant difference. (F) MiR-122 inhibits phosphorylation of PKR (p-PKR). Lysates from transfected LX-2 cells were immunoblotted with antibodies against p-PKR (Thr451), PKR or GAPDH. GAPDH was used as internal control. Minimum three replicates were performed for each set of experiments to compile the data as presented.

To validate whether miR-122 inhibits PACT mRNA expression, we performed real-time PCR analysis of LX-2 cells transfected with miR-122 or miR-C. We confirmed the significant down-regulation of PACT mRNA expression in the LX-2 cells transfected with miR-122 (Fig 4A).

To examine whether miR-122 inhibits PACT expression at the protein level, we also performed western blot analysis of LX-2 cells transfected with miR-122 or miR-C. A significant down-regulation of PACT expression in the LX-2 cells expressing miR-122 was observed at the protein level (Fig 4B).

To confirm that miR-122 directly regulates PACT expression, we examined whether its overexpression inhibited the activity of a luciferase reporter construct containing PACT 3´-UTR (wild) or PACT 3´-UTR (mutant) (Fig 4C). MiR-122 reduced the luciferase activity of PACT 3´-UTR (wild) (Fig 4D) but not the PACT 3´-UTR (mutant) (Fig 4E), suggesting that the mutation in the seed sequence prevented the binding of miR-122 to 3´-UTR (Fig 4C). Moreover, we observed a reduction in the phosphorylation status of PKR in miR-122-transfected LX-2 (Fig 4F). Taken together, these data demonstrate that PACT is a direct target of miR-122.

### Knockdown of PACT inhibits cytokine production in LX-2 cells

**Fig 5:**
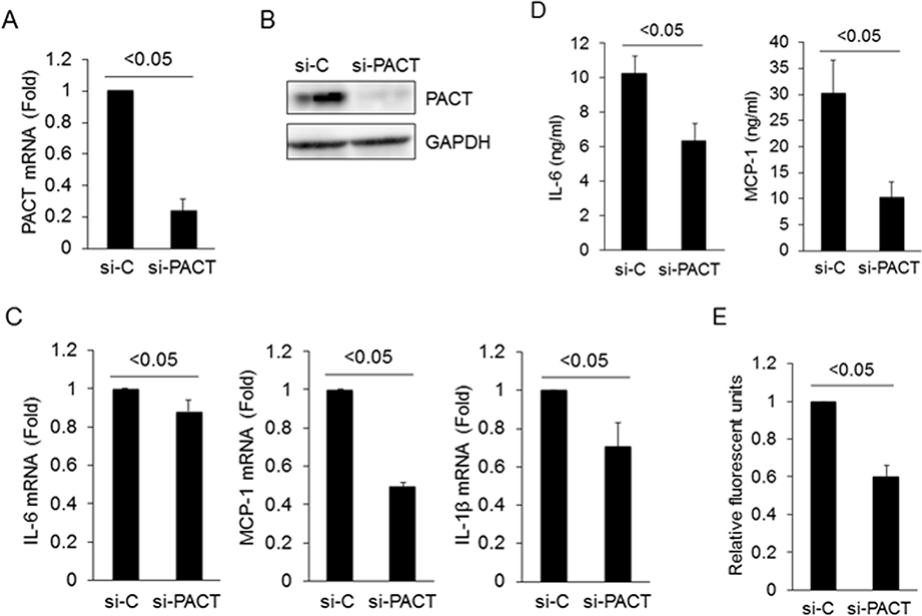
Knockdown of PACT inhibits cytokine production in LX-2 cells and suppresses migration of THP-1 cells. (A) Knockdown of PACT mRNA expression in LX-2 cells. LX-2 cells were transfected with 20 nM control siRNA (si-C) or 20 nM siRNA against PACT (si-PACT). After incubation in DMEM with 1% FCS for 24 hours, the cells were treated with 100 ng/mL LPS for 24 hours. Cellular RNAs were isolated, and PACT mRNA was examined by real-time RT-PCR. GAPDH was used for normalization. All results are shown as mean ± SD. (B) Knockdown of PACT protein expression in LX-2 cells. Lysates from transfected LX-2 cells were immunoblotted with antibodies against PACT or GAPDH. GAPDH was used as internal control. (C) Effect of PACT knockdown on mRNA expression of cytokines. Cellular RNA was isolated from LPS-stimulated LX-2 cells, and the mRNA expression levels of IL-6, MCP-1 and IL-1β mRNA were examined by real-time RT-PCR. GAPDH was used for normalization. (D) Effect of PACT knockdown on protein expression of cytokines. Conditioned medium from LPS-stimulated LX-2 cells was collected, and ELISA was performed to assess the expression of IL-6 and MCP-1. (E) Migration of THP-1 cells was suppressed by conditioned medium from si-PACT-transfected LX-2 cells. A total of 5 x 10^5^ THP-1 cells were placed into the upper chamber, and conditioned medium from LPS-stimulated LX-2 cells transfected with si-C or si-PACT was added to the lower chamber. After 3 hours of incubation, migration of THP-1 cells toward the lower chamber was detected with a fluorescent dye. Relative fluorescence units are indicated (vs. control si-C). All results are shown as mean ± SD. Minimum three replicates were performed for each set of experiments to compile the data as presented.

After the specificites of si-PACT and si-C were validated (Fig 5A and 5B), the expression levels of IL-6, MCP-1 and IL-1β mRNA in LX-2 cells transfected with si-PACT or si-C were analyzed using real-time RT-PCR. Knockdown of PACT significantly reduced the expression levels of IL-6, MCP-1 and IL-1β mRNAs (Fig 5C). The effects of the knockdown of PACT on IL-6 and MCP-1 expression were also confirmed by ELISA. The protein levels of IL-6 and MCP-1 were significantly reduced in the conditioned medium from the si-PACT-transfected LX-2 cells (Fig 5D). IL-1β in the conditioned medium from the untransfected, si-C-transfected, or si-PACT-transfected LX-2 cells was undetectable, 6.28 pg/mL, or undetectable, respectively.

### Conditioned media from si-PACT-transfected LX-2 cells suppress human monocyte-derived THP-1 cell migration

We confirmed that the knockdown of PACT reduced IL-6, MCP-1, and IL-1β expression in LX-2 cells. Next, we examined the effects of PACT knockdown on monocyte migration by *in vitro* migration assays. For this experiment, conditioned medium from LX-2 cells transfected with si-C or si-PACT was evaluated. The migration of THP-1 cells was markedly decreased (~60%) when conditioned medium from LX-2 cells transfected with si-PACT was used (Fig 5E). The number of migrated THP-1 cells was 37,300/well, 42,900/well or 25,900/well when conditioned medium from LX-2 cells transfected with mock, si-C or si-PACT, respectively, was used. These data suggest that the inhibition of inflammatory cytokine production together with the knockdown of PACT in HSCs inhibits the migration of monocytes and monocyte-derived cells, supporting the notion that miR-122 inhibits cytokine production in HSCs and the recruitment of immune cells to the liver at least in part, due to the suppression of PACT.

### Inhibition of PKR suppresses cytokine production in LX-2 cells

**Fig 6:**
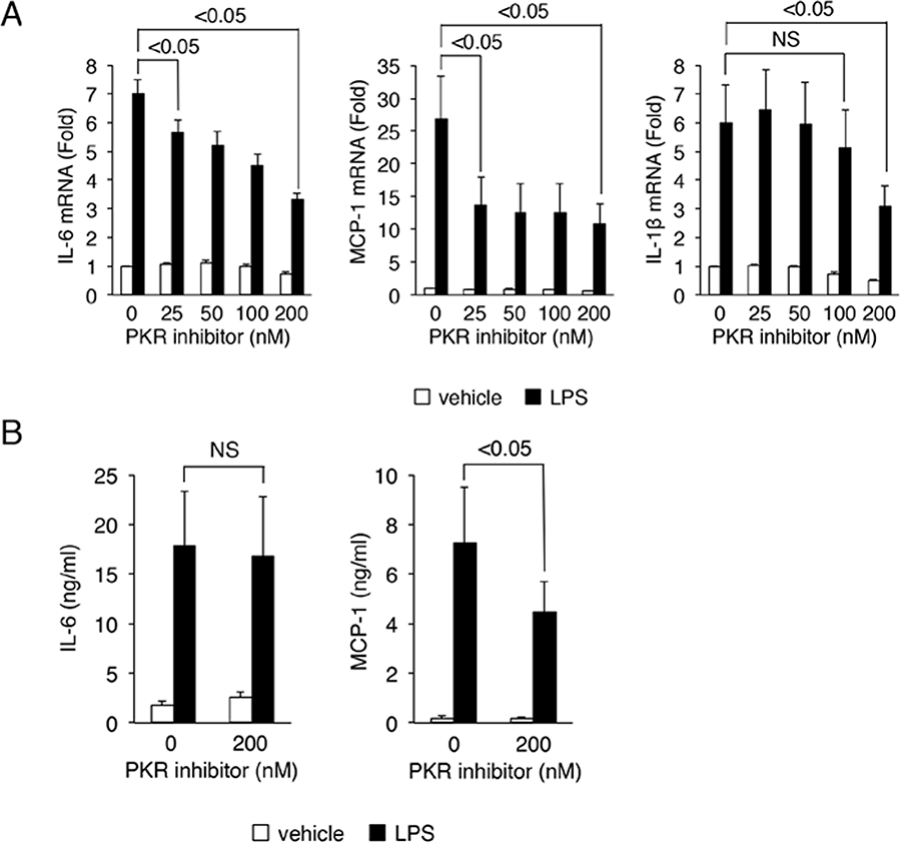
Inhibition of PKR suppresses cytokine production in LX-2 cells. The PKR inhibitor C16 or a vehicle was added to LX-2 cells at 1 hour prior to LPS stimulation at the indicated concentrations. Then, the cells were treated with 100 ng/mL LPS for 24 hours in the presence or absence of the PKR inhibitor C16. (A) Effects of inhibition of PKR on expression of cytokine mRNA. Cellular RNA was collected, and the mRNA levels of IL-6, MCP-1 and IL-1β were examined by real-time RT-PCR. GAPDH was used for normalization. (B) Effects of inhibition of PKR on expression of cytokines at protein level. IL-6 and MCP-1 protein levels were determined by ELISA. The results are expressed as mean ± SD. Minimum three replicates were performed for each set of experiments to compile the data as presented. Black and white columns indicate presence and absence of the PKR inhibitor C16, respectively.

PACT was originally identified as a protein activator of PKR [12–14]. We also assessed the effect of the pharmacological inhibition of PKR activity on cytokine production in LX-2 cells. In LX-2 cells treated with LPS and the PKR inhibitor C16, we observed the down-regulation of IL-6, MCP-1 and IL-1β mRNA in a dose-dependent manner (Fig 6A). We also confirmed the down-regulation of MCP-1 protein by PKR inhibitor C16 in the presence of LPS stimulation by ELISA (Fig 6B). MTS assay showed that the PKR inhibitor C16 had no effect on cell viability at the concentration used in this experiment. We expected that miR-122 might inhibit PACT expression and the interaction between PACT and PKR. Further study will be needed to clarify this issue.

### MiR-122 blocks NF-κB nuclear translocation in LX-2 cells

**Fig 7:**
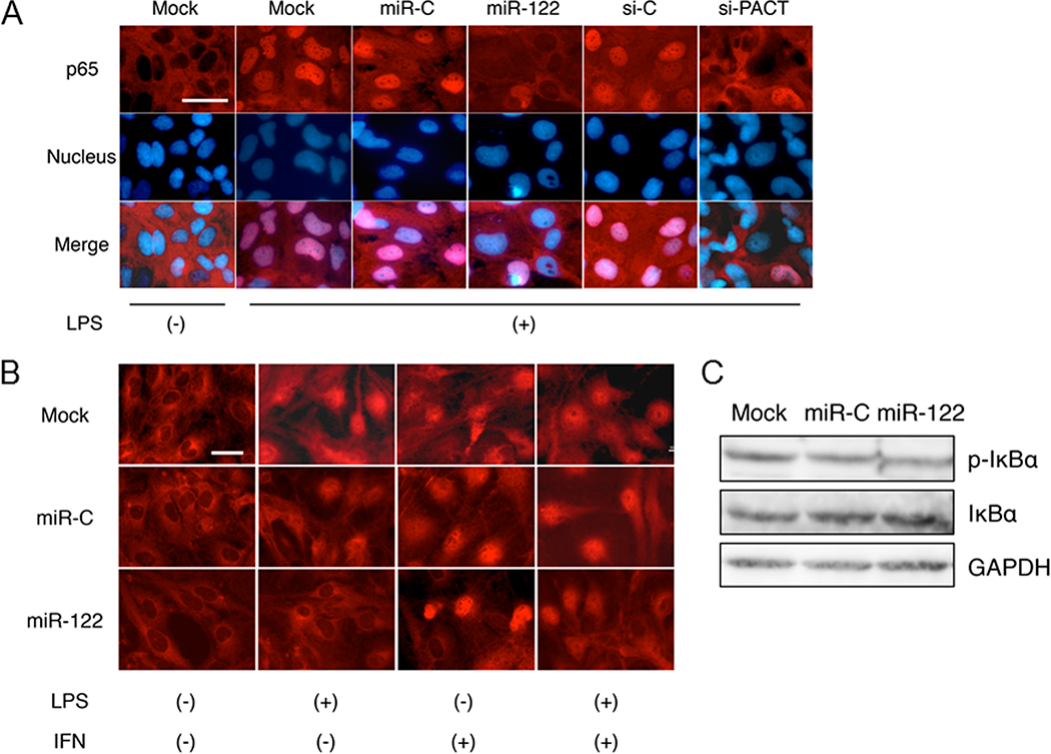
NF-κB nuclear translocation decreases in LX-2 cells transfected with miR-122 or si-PACT. (A) LX-2 cells were transfected with miR-C, miR-122, si-C or si-PACT. Thirty-six hours post-transfection, the cells were treated with 100 ng/mL LPS. Two hours later, they were fixed and stained with an antibody against NF-κB p65 (red). Nuclei were visualized by staining with Hoechst 33342 (blue). Scale bar: 25μm. (B) Interferon-α treatment partially rescued NF-κB nuclear localization blocked by miR-122. Cells were fixed and stained with an antibody against NF-κB p65 (red). Scale bar: 25μm. (C) MiR-122 inhibits phosphorylation of IκBα (p-IκBα). Lysates from transfected LX-2 cells were immunoblotted with antibodies against p-IκBα (Ser32), IκBα or GAPDH. GAPDH was used as internal control. Minimum three replicates were performed for each set of experiments to compile the data as presented.

NF-κB is an important regulator of immunity and inflammation [30,31]. It is sequestered in an inactive form in the cytoplasm, where it is bound to inhibitory IκB proteins [31]. The binding of LPS to TLR4 leads to rapid phosphorylation, ubiquitinylation and the proteolytic degradation of IκB, which frees NF-κB to translocate to nucleus, resulting in the activation of the transcription of its target genes, such as inflammatory cytokines and chemokines. We also postulated that indirect inhibition of the activation of NF-κB by miR-122 might result in inhibition of cytokine production, as this transcription factor plays a role in LPS-induced inflammatory cytokine production [7,24]. In this study, we examined the sublocalization of NF-κB in LX-2 cells transfected with miR-122 or si-PACT after LPS stimulation (Fig 7A). In mock-transfected, miR-C-transfected and si-C-transfected cells, NF-κB was predominantly localized to the nucleus, a sign of NF-κB activation (Fig 7A). However, NF-κB-nuclear translocation was observed less frequently in LX-2 cells transfected with miR-122 or si-PACT, compared with that in LX-2 cells transfected with miR-C or si-C (Fig 7A). These findings suggest that miR-122 can affect cytokine production, at least in part, through NF-κB. We also co-expressed miR-122 and PKR induced by IFN-α in LX-2 cells, in the presence of LPS stimulation, and observed that NF-κB nuclear localization was partly rescued (Fig 7B). We also observed that miR-122 inhibited the phosphorylation of IκBα (Fig 7C).

## Discussion

Small noncoding miRs regulate diverse biological functions in the liver and play roles in liver pathology and liver diseases. Down-regulation of miR-122 in the liver has been reported in certain patients with advanced liver disease and in animal models of hepatic fibrosis. In the present study, we described at least four important findings, demonstrating that miR-122 can affect HSCs and that it is involved in immune response in the liver, including the following: 1) miR-122 negatively regulates the production of IL-6, MCP-1 and IL-1β after LPS stimulation in HSCs (Fig 2); 2) miR-122 directly targets the PKR activator, PACT (Fig 4); 3) PKR signaling can also play a role in cytokine production in HSCs, at least in part through NF-κB (Figs 6 and 7); and 4) Conditioned media from miR-122-transfected LX-2 cells can suppress the migration of human monocyte-derived THP1 cells, suggesting that this miR affects the migration of monocytes and monocyte-derived cells in the liver (Fig 2). Taken together, miR-122 could prevent hepatic inflammation by inhibiting LPS-induced cytokine production and the recruitment of immune cells to the liver.

Chronic inflammation of the liver is observed in various chronic liver diseases, such as chronic hepatitis C and B, autoimmune liver diseases, ALD and NASH [32–34]. Chronic hepatic inflammation results in hepatic fibrosis and cirrhosis [35]. Gut-derived LPS-activated TLR4 signaling also contributes to fibrosis of the liver [7]. HSCs play roles in inflammation as well as fibrosis of the liver [1,2]. Seki et al. [36] have reported that TLR4 signaling in HSCs promotes cytokine production, induces chemotaxis of macrophages and downregulates the TGF-β pseudo-receptor Bambi to sensitize HSCs to TGF-β signaling through an MyD88-NF-κB-dependent pathway. In the present study, miR-122 inhibited TLR4 signaling after LPS stimulation in HSCs, indicating that it may play an important role in preventing liver inflammation caused by bacterial translocation.

Liver inflammation is regulated by cytokines and chemokines, which control the migration and activities of hepatocytes, Kupffer cells, HSCs, endothelial cells and circulating immune cells [29]. MCP-1 is a well-characterized chemokine involved in hepatic inflammation. It seems to play a role in steatohepatitis in ALD and NASH model mice [37,38] and promotes hepatic fibrosis [39] by recruiting monocytes to the liver. Kupffer cells, injured hepatocytes and activated HSCs secrete high levels of MCP-1 [29,36]. MiR-122-deficient mice show increased hepatic inflammation through the up-regulation of MCP-1 in hepatocytes, directly by the targeting of this transcript and indirectly as in a response to underlying hepatocyte injury [19]. In the present study, we showed another mechanism of MCP-1 inhibition by miR-122, suggesting the importance of HSCs in hepatic inflammation.

We and others [25] have identified PACT as one of the target genes of miR-122. We confirmed that 8-nt seed matches seem to result in the regulation of a given message by miR-122 (Fig 4C). Furthermore, we observed that the down-regulation of PACT by miR-122 and the pharmacological inhibition of PKR activity suppressed cytokine production in HSCs, suggesting the importance of PKR signaling. PACT is activated by bacterial products and cellular stress [40]. Yoshida et al. [41] have reported that PACT-PKR interaction and phosphorylation of PKR are associated with the induction of NF-κB by LPS. Treatment of PKR inhibitor C16 prevented activation of NF-κB [42]. LPS-induced PACT activation and PACT-PKR interaction may play important roles in cytokine production in HSCs. PKR and PKR-like endoplasmic reticulum kinase (PERK) phosphorylate eIF2α. Sustained eIF2α phosphorylation, a hallmark of unfolded protein response in cirrhotic liver, is associated with a lack of LPS-induced accumulation of NF-κB-dependent anti-apoptotic proteins, which may sensitize cirrhotic livers to LPS/TNFα-mediated apoptosis [43]. In advanced liver disease, PKR seems to be an important treatment target.

There have been several reports of the association of the down-regulation of hepatic miR-122 with human liver diseases. A reduction in hepatic miR-122 expression has been observed in patients with NASH and chronic hepatitis B infection [44,45]. In chronic hepatitis C patients with advanced fibrosis, hepatic miR-122 is also decreased [46,47]. Although this miR is abundantly expressed in hepatocytes, it is also expressed in HSCs. In addition, because exosomes, nano-sized membranous vesicles, have been reported in association with the transfer of miRs between hepatic cells [48], it is possible that miR-122 derived from hepatocytes affects the functioning of HSCs. Therefore, the down-regulation of miR-122 in hepatocytes may also increase cytokine production in these cells and contribute to the progression of liver disease. In humans, changes in the level of miR-122 in HSCs during the progression of chronic liver disease and the influence of this miR on hepatic inflammation in these cells have not been well assessed. Further study is needed in this regard.

Previous reports have shown that transcription factors, such as CCAAT/enhancer-binding protein α (C/EBPα) and hepatocyte nuclear factor 4α (HNF4α), promote miR-122 expression in hepatocytes [49] and HSCs [20]. Song et al. [50] have shown that peroxisome proliferator-activated receptor γ (PPARγ) promotes miR-122 transcription and that hepatitis B virus X protein inhibits PPARγ binding. However, the mechanism of the regulation of miR-122 is still not well understood. Further study concerning the regulation of miR-122 may also be important for the increased understanding of chronic liver disease.

PMA-stimulated THP-1 cells were used as one of the types of macrophage-like cells [51]. We observed that miR-122 inhibited the migration of PMA-stimulated THP-1 cells more weakly than PMA-unstimulated THP-1 cells (~3-fold vs. 1.1-fold). A different mechanism of migration between monocytes and macrophages may exist [52]. Previous study has shown that miR-122 expression levels in primary HSCs are ~25% of those in primary hepatocytes [20]. Similarly, miR-122 expression levels in LX-2 cells are ~20% of those in Huh7 cells [20]. Further, real-time PCR assay showed no miR-122 expression in THP-1 cells under our experimental conditions. Although monocytes are the main cell types to produce inflammatory cytokines inside the liver, we chose LX-2 cells and conducted experiments with these cells in the present study. The amount of miR-122 mimic used in the present study seems high, but it was similar to previous reports [20,53,54]. It was reported that miR-122 levels go down with fibrosis and also in activated HSCs [44–46]. Further studies may be needed to examine whether overexpression of miR-122 can also inhibit inflammatory cytokine production in monocytes.

HSCs may not be the major source of liver inflammation, and they produce miR-122 at low levels. However, Momen-Heravi et al. [55] have recently shown that exosomes mediated communication between hepatocytes and monocytes/macrophages, and that hepatocyte-derived miR-122 could reprogram monocytes, inducing sensitization to LPS. So, we also focused on the LPS and TLR4 systems in LX-2 cells. It is known that LPS treatment upregulates PACT expression [41], but we did not observe any differences in miR-122 expression between HSCs treated with or without LPS (1.1 or 1.0-fold, respectively).

We observed that IFN treatment in miR-122 overexpressed LX-2 cells treated with LPS rescue NF-κB nuclear translocation (Fig 7B). These results also supported the previous observation that IFN could modulate the expression of liver-specific miR-122 [56], which may be due to the fact that IFN treatment can inhibit miR-122 expression and induce PACT expression. In the present study, we observed that the overexpression of miR-122 could inhibit the production of inflammatory cytokines and migration of monocytes. Further *in vivo* studies may be needed to elucidate this point. In conclusion, our study demonstrated that miR-122 downregulates cytokine production in HSCs and inhibits monocyte chemotaxis. The targeting of miR-122 may shed new light on therapeutic options to prevent the progression of liver diseases.

## Abbreviations

IL: interleukin
MCP-1: monocyte chemoattractant protein-1
CCL2: chemokine (C-C motif) ligand 2
PACT: PKR-activating protein;
CSF2: colony-stimulating factor 2
SARM1: sterile alpha and TIR motif containing 1
REL: V-rel reticuloendotheliosis viral oncogene homolog
Fos: FBJ murine osteosarcoma viral oncogene homolog
IRF1: interferon regulatory factor 1
HMGB1: high mobility group box 1
RIPK2: receptor-interacting serine-threonine kinase 2
NFKB1A: nuclear factor of kappa light polypeptide gene enhancer in B cells inhibitor alpha
IRAK: interleukin-1 receptor-associated kinase
ELK1: member of the ETS oncogene family
NR2C2: nuclear receptor subfamily 2, group C, member 2
MAP3K: mitogen-activated protein kinase kinase kinase
PTGS2: prostaglandin-endoperoxide synthase 2
JUN: Jun proto-oncogene
FADD: Fas-associated via death domain
MAPK: mitogen-activated protein kinase
EIF2AK2: eukaryotic translation initiation factor 2-alpha kinase 2
TLR: Toll-like receptor
PPARA: peroxisome proliferator-activated receptor alpha
LY96: lymphocyte antigen 96
MYD88: myeloid differentiation primary response gene (88)
IFNA1: interferon, alpha 1
TNF: tumor necrosis factor
CXCL10: chemokine (C-X-C motif) ligand 10
CD180: CD180 molecule
